# Ondansetron induced *torsades de pointes*

**DOI:** 10.1002/ccr3.2251

**Published:** 2019-07-07

**Authors:** Eshan Patel, Danielle Rosemond, Ashwad Afzal

**Affiliations:** ^1^ Department of Medicine, NewYork‐Presbyterian Brooklyn Methodist Hospital Affiliate of Weill Medical College of Cornell University Brooklyn New York

**Keywords:** antiemetic, drug‐induced torsades, Ondansetron, QT prolongation, torsades de pointes

## Abstract

A single dose of oral Ondansetron can precipitate torsades de pointes and other arrhythmias in patients with risk factors that may prolong QTc interval.

A 60‐year‐old woman presented with nausea, vomiting, and abdominal pain for 1 day. Past medical history included gastritis and sinus bradycardia with first‐degree atrioventricular block. Physical examination was pertinent for bradycardia at 46 beats per minute (bpm) and mild epigastric tenderness. Chest radiograph and laboratory evaluation were normal with the exception of magnesium level 1.7 mmol/L. Electrocardiogram (ECG) showed sinus bradycardia 37 bpm, first‐degree AV block, QTc 408 ms. Patient had an episode of nausea that was treated with Ondansetron 4 mg orally disintegrating tablet resulting in improvement. Two hours after, telemetry monitor revealed sinus bradycardia ranging from 37‐45 bpm with episodes of ventricular tachycardia. An ECG revealed sinus bradycardia, first‐degree AV block, premature ventricular contraction followed by fusion of T wave on U wave leading to polymorphic ventricular tachycardia with beat‐to‐beat variations evident of *torsades de pointes* (Figure 1). Urgent treatment with magnesium sulfate 2 g IV and oral magnesium oxide 400 mg was successful in terminating the arrhythmia.

**Figure 1:**
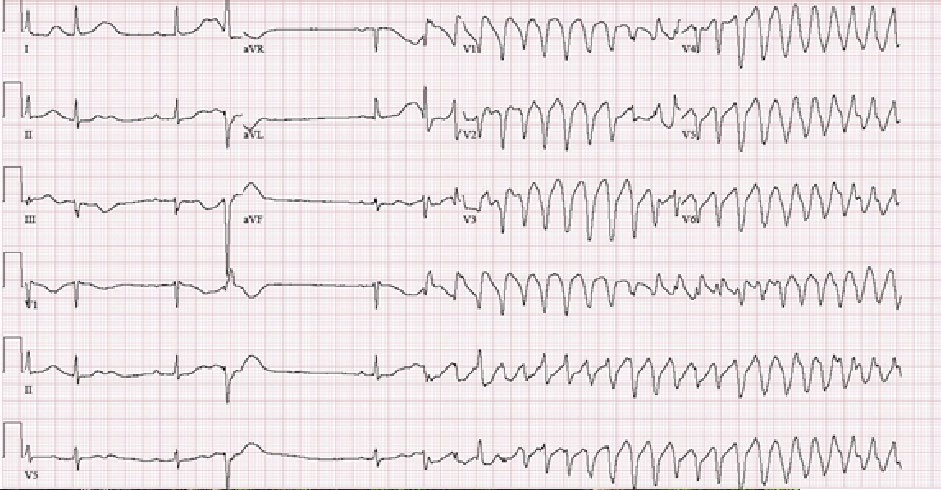
ECG showing sinus bradycardia, first‐degree AV block, premature ventricular contraction followed by fusion of T wave on U wave leading to polymorphic ventricular tachycardia with beat‐to beat variations evident of torsades de pointes

Risk factors for developing *torsades de pointes* include cardiac diseases, congenital long QT syndrome, female gender, bradycardia, hypothermia, hypomagnesemia, hypokalemia and medications that may cause QT prolongation.^1^ Given our patient's risk factors, the addition of Ondansetron likely triggered her torsade de pointe.

## CONFLICT OF INTEREST

None declared.

## AUTHOR CONTRIBUTIONS

EP: acquired data and drafted the initial manuscript. DR: revised manuscript. AA: interpreted ECG and revised manuscript.
